# Determinants of stunting, underweight and wasting among children < 5 years of age: evidence from 2012-2013 Pakistan demographic and health survey

**DOI:** 10.1186/s12889-019-6688-2

**Published:** 2019-04-01

**Authors:** Sadaf Khan, Sidra Zaheer, Nilofer Fatimi Safdar

**Affiliations:** 1 Department of Biochemistry, Dow International Medical College, Dow University of Health Sciences, Karachi, Pakistan; 2 Dow Research Institute of Biotechnology and Biomedical Sciences, Dow University of Health Sciences, Karachi, Pakistan; 3 School of Public Health, Dow University of Health Sciences, Karachi, Pakistan

**Keywords:** Malnutrition, Stunting, Wasting, Underweight, Children

## Abstract

**Background:**

Childhood malnutrition is a critical public health concern in Pakistan. We aimed to explore factors associated with malnutrition in Pakistani children (< 5 years of age) using the Pakistan Demographic and Health Survey (PDHS) 2012–2013.

**Methods:**

Sample of 3071 Pakistani children aged 0–59 months from the PDHS 2012–2013, with complete anthropometric measurements were included in the study. Nutritional status was evaluated using anthropometric indices; height-for-age, weight-for-height and weight-for-age, as proxy measures of three forms of under-five malnutrition including stunting, wasting and underweight respectively. Uni- and multivariate binary logistic regressions were used to examine the association between selected maternal-socio-demographic and child level variables (such as child sex, age, size at birth, antenatal clinic visits, recent diarrheal incidence and breastfeeding status) and three proxy measures of child nutritional status.

**Results:**

About 44.4% of under-five children were stunted, 29.4% were underweight and 10.7% were wasted. Children whose mothers lived in rural areas (aOR = 0.67, 95%CI 0.48–0.92), were aged ≥18 years at marriage (aOR = 0.76, 95%CI 0.59–0.99) and had visited antenatal clinic more than 3 times during pregnancy (aOR = 0.61, 95%CI 0.38–0.98) were less likely to be stunted. Mother’s low educational level (aOR = 2.55, 95%CI 1.26–5.17), short stature (aOR = 2.31, 95%CI 1.34–3.98), child’s small size at birth (aOR = 1.67, 95%CI 1.14–2.45) and mother’s BMI were significantly associated with child’s underweight status. Children whose mothers had no education were more likely to be wasted (aOR = 3.61, 95%CI 1.33–9.82).

**Conclusion:**

The study suggests that most of the analysed factors that accounted for malnutrition in Pakistani children (such as mother’s age at marriage, educational level and mothers’ nutritional status) are preventable. Therefore, to reduce the burden of malnutrition interventions that can address these factors are required such as community based education and targeted nutritional interventions.

## Background

Malnutrition remains a critical public health problem among children under the age of five years in developing countries including Pakistan. Malnutrition is caused by multiple interlinked factors and has both short and long term detrimental health effects [1, 2]. It affects the cognitive and physical development of children, increases the risk of infections and significantly contributes to the child’s morbidity and mortality [3, 4]. Stunting, wasting and underweight are three widely recognized indicators of child's nutritional status [5]. While stunting and wasting indicates chronic and acute malnutrition respectively, underweight is a composite indicator and includes both acute (wasting) and chronic (stunting) malnutrition [5]. However, different forms of malnutrition can also occur concurrently in children [6].

Malnutrition significantly contributes to the global burden of several diseases. Globally, undernutrition accounts for at least half of all the deaths annually in children under five [7]. In 2016, according to World Health Organization (WHO), at least 155, 52 and 99 million children under the age of five years were stunted, wasted and underweight worldwide respectively [8, 9]. In addition, around 6 million children were reported with stunting and wasting simultaneously [6]. Malnutrition is clustered in developing countries, particularly in Africa and South Asia [9, 10]. In South Asia, three countries of the region, India, Pakistan and Bangladesh, have particularly high prevalence of the condition [11].

In Pakistan, malnutrition is the major contributor of morbidity and mortality in children under five years of age, and the country ranks 22nd in the world for under-five child mortality [12, 13]. The 2011 national nutrition survey reported that 44% of under-five children in Pakistan were stunted, 15% were wasted and 31% were underweight [14]. The high magnitude of all three indicators of malnutrition in country reflects the poor nutritional and health status among under-fives in country, thus necessitating the need to conduct this study to explore factors associated with malnutrition in Pakistani children.

There are multiple factors that contribute to childhood malnutrition. The common determinants reported by several studies include socioeconomic inequalities, geographical differences, suboptimal feeding practices, household food insecurity, maternal literacy and childhood morbidities [15–19]. Previous studies that have been conducted on childhood malnutrition in Pakistan were mostly based on hospital, schools, regional and community settings [20–23]. Limited studies, that have reported the national level data, were either restricted to socio-demographic determinants or children less than two years of age [24, 25]. In addition, there is a paucity of literature regarding the correlates of all three indicators of childhood malnutrition in the country based on nationally representative data set. Therefore, the current study utilized the nationally representative Pakistan Demographic and Health Survey (PDHS) 2012–2013 data, to examine the factors associated with malnutrition among Pakistani children aged 0–59 months.

## Methods

### Study design

Cross-sectional.

### Data set

Secondary analysis of the PDHS 2012–2013 data set was carried out. The PDHS 2012–2013 was a nationally representative third survey conducted by the National Institute of Population Studies (NIPS), Pakistan, as a part of the international MEASURE DHS (Demographic and Health Surveys) program. The survey was carried out with the support of ICF International and United States Agency for International Development (USAID) [26]. This survey collected the information of Pakistani households related to socio-demographic, maternal and child health indicators [26].

### Study sampling and participants

A two stage stratified sampling design was used in order to obtain a nationally representative sample of Pakistani households for the PDHS 2012–2013. The survey included the rural and urban population of all four provinces of Pakistan (Punjab, Sindh, Khyber Pakhtunkhwa and Balochistan) and regions of Gilgit Baltistan and Islamabad Capital Territory (ICT). However, the survey did not collect the data from some parts of the country including Federally Administrated Tribal Areas (FATA), Azad Jammu and Kashmir (AJK) and restricted military areas due to security concerns [26]. For the purpose of the PDHS 2012–2013, 13,944 households were selected and interviews were successfully completed in 96% households by trained interviewers using the structurally validated questionnaire. Women who were ever married (*n* = 14,569, age 15–49 years) were interviewed and anthropometric measurements of eligible children (*n* = 3466) were recorded from a subset of these households. Eligible children included were born within the five years preceding the survey and had valid record of dates of birth. Measurements of children were recorded for both height (in centimeter) and weight (in kilograms) using the digital SECA scales and measuring boards of Shorr productions. Recumbent length was measured in children who were either less than 2 years of age or 85 cm in length and for the rest standing height was measured [26].

The sample of children (*n* = 3071, aged 0–59 months) that had complete anthropometric measurements were selected from the subset of eligible children (*n* = 3466) for the current analysis.

### Evaluation of nutritional status of children < 5 years of age

We used three widely recognized anthropometric indices (height-for- age, weight-for-height and weight-for-age) to assess the nutritional status of under-five children in the PDHS 2012–2013 data set. The WHO Multicenter Growth Reference Study was used to calculate the three anthropometric indices in order to evaluate the nutritional status [27]. The three indices were expressed in standard deviation (SD) units from reference population median. Children with Z-scores, below -2SD from the WHO reference population median, for height-for-age (HAZ), weight-for-height (WHZ) and weight-for-age (WAZ) were considered stunted, wasted and underweight respectively [26].

### Study variables

The coding plan of the selected study variables is given in Table 1. To examine the factors linked with nutritional status of the children less than 5 years of age, dependent variables were expressed as dichotomous variables. The variables included category 0 [not stunted (> − 2SD), not wasted (> − 2SD to +2SD) and not underweight (> − 2SD)] and category 1 [stunted (< − 2SD), wasted (< − 2SD) and underweight (< − 2SD)]. Obese children (WHZ above +2SD, *n* = 208) were not included in the analysis of wasted children (Table 2).

**Table 1:** Description and analysis coding plan of the selected study variables

Variables	Description and Categorization	Analysis Coding
Outcome variable		
Stunting (Height-for-age index)	Height/Age standard deviation (new WHO) (Continuous)	0 = Normal height-for-age/Not-stunted (HAZ -2SD and Above) 1 = Stunted (HAZ < -2SD)
Underweight (Weight-for age index)	Weight/Age standard deviation (new WHO) (Continuous)	0 = Normal weight-for-age/Not-underweight (WAZ-2SD and Above) 1 = Underweight (WAZ < -2SD)
Wasting (Weight-for-height index)	Weight/Height standard deviation (new WHO) (Continuous)	0 = Normal weight-for-height/Not-wasted (WHZ-2SD to +2SD) 1 = Wasted (WHZ < -2SD)
Independent variables		
Residence	Type of place of residence (1 = Urban,2 = Rural)	Used same coding for analysis
Mother’s educational level	Highest educational level (0 = no education,1 = primary,2 = secondary,3 = higher)	Used same coding for analysis
Wealth index	Wealth index (1 = poorest, 2 = poorer, 3 = middle, 4 = richer, 5 = richest)	Used same coding for analysis
Mother’s employment status	Respondent currently working (0 = Non-working, 1 = Working)	Used same coding for analysis
Mother’s access to information	1. Frequency of reading newspaper or magazine 2. Frequency of listening to radio 3. Frequency of watching television (0 = Not at all, 1 = Occasionally, 2 = Atleast once a week, 3 = Daily)	Options for all the three variables were recoded as follows 0 as 0 = No (No Access) 1,2&3 as 1 = Yes (Access to at least one of the information source)
Consanguineous marriage	Blood relation with husband (0 = No, 1 = Yes)	Used same coding for analysis
Mother’s age at marriage	Age at first cohabitation (Continuous,Range:10–49)	Constructed as 1 = < 18 2= > 18
Parity	Total children ever born (Continuous,Range:1–19)	Constructed as 1 = 1–4 2= > 4 (5+)
Mother’s height (cm)	Respondent’s height in centimeters (1 decimal)(Continuous)	Constructed as follows 1 = Normal (>145 cm) 2 = Short Stature (< 145 cm)
Mother’s BMI (kg/m^2^)	Body Mass Index (Continuous, calculated using measured height and weight)	1 = Underweight (<18.5 kg/m^2^) 2 = Normal (18.5–24.9 kg/m^2^) 3 = Overweight (25.0–29.9 kg/m^2^) 4 = Obese (≥ 30 kg/m^2^)
Age of Child (years)	Current age of the child (Continuous)	Constructed as 0 = < 12 months 1 = 12–23 months 2 = 24–35 months 3 = 36–47 months 4 = 48–59 months
Sex of child	Sex of child (1 = Male, 2 = Female)	Used same coding for analysis
Child size at birth	Size of child at birth (1 = very large,2 = larger than average,3 = average,4 = smaller than average,5 = very small,6 = Don’t know)	Options were recoded as follows 4 & 5 as 1 = smaller than average 3 & 6 as 2 = average 1 &2 as 3 = larger than average
Antenatal clinic visits	Number of antenatal visits during pregnancy (0 = No antenatal visit, 98 = Don’t know)	Constructed as follows 0 & 98 as 0 = No Visit 1–3 as 1 = Less frequent visits Visit frequency > 3 as 2 = Frequent visits (3+)
Recent diarrheal incidence (last two weeks)	Had diarrhea recently (0 = No,1 = Yes, last 24 h,2 = Yes, last 2 weeks, 8 = Don’t Know)	Recoded as 0as 0 = No 1&2as1 = Yes 8 = Excluded
Breastfeeding status	Duration of breastfeeding (93 = Ever breastfeeding, 94 = Never breastfeed, 95 = Still breastfeeding, 96 = Breastfed until died,97 = Inconsistent, 98 = Don’t know)	Used same coding for analysis (No data were found for 96,97,98)

**Table 2:** Prevalence of different forms of malnutrition in Pakistani children aged 0-59 months (*n* = 3071)

Variable	N	Weighted %	Mean (SD)
Stunting (HAZ)			
-2SD and above (Normal/Non-stunted)	1691	55.59	−0.57 ± 0.04
Below -2SD (Moderately stunted)	587	21.14	− 2.48 ± 0.01
Below -3SD (Severely stunted)	793	23.27	−3.97 ± 0.03
Mean Z score (SD)			−1.77 ± 0.04
Underweight (WAZ)			
-2SD and above (Normal/Not-underweight)	2255	70.62	−0.77 ± 0.03
Below -2SD (Moderately underweight)	549	19.97	− 2.46 ± 0.02
Below -3SD (Severely underweight)	267	9.41	−3.68 ± 0.05
Mean Z score (SD)			−1.38 ± 0.04
Wasting (WHZ)			
Above +2SD (obese)	208	3.27	2.84 ± 0.09
-2SD to +2SD (Normal/Not-wasted	2544	86.05	−0.35 ± 0.02
Below -2SD (Moderately wasted)	217	7.56	− 2.34 ± 0.02
Below -3SD (Severely wasted)	102	3.12	−3.68 ± 0.06
Mean Z score (SD)			−0.50 ± 0.04

Stunted, underweight and wasted children include both moderately and severely stunted, underweight and wasted children respectively. Obese (WHZ above +2SD) were not included in the wasted children analysis

Explanatory variables were selected after conducting a detailed literature review [15–17, 19, 23, 24, 28] and only those variables showing association with nutritional status of children and also available with complete information in the PDHS 2012–2013 data set were included in the current analysis. Selected explanatory variables were divided into two levels which included sociodemographic-maternal and child-level factors. Socio demographic-maternal factors selected were types of residence, household wealth index, mother’s educational level, employment status, age at marriage, parity, access to information, consanguineous marriage, mother’s height and mother’s body mass index (BMI). Child-level factors were sex of child, child age, child size at birth, antenatal clinic visits, recent diarrheal incidence and breastfeeding status.

### Statistical analysis

We used software SPSS 16.0 and SAS 9.1 for data analysis. Analysis was done by descriptive statistics and logistic regressions. Descriptive statistics were used to generate frequencies and to describe the study variables. Univariate and multivariate binary logistic regressions were used to examine the determinants of all three indices of child nutritional status. Prevalence of nutritional status in the population and all the regression analysis models were adjusted to consider the complex sampling design of the PDHS 2012–2013. The adjustment was made by including the primary sampling units, final weights and strata in the models.

Two step wise models were constructed for the study, based on the categorization of the independent variables into sociodemographic-maternal and child level factors. Model 0 reported the univariate association between child nutritional status and all independent study variables. Model 1 showed adjusted associations after including type of residence, wealth index, mother’s education level, employment status, age at marriage, parity, access to information, consanguineous marriage, mother’s height and BMI. Model 2 reported the results after adjusting for child level factors which includes child age, sex, perceived child weight at birth, antenatal clinic visits, recent diarrheal incidence, and breastfeeding status. Results were given as crude odds ratios (cOR) and adjusted odds ratios (aOR) with 95% confidence intervals (CI). *P*-values < 0.05 were considered statistically significant.

### Ethical review

The PDHS 2012–2013 has taken into account the standard ethical guidelines of the measure DHS program [26]. The authors have obtained the data from MEASURE DHS website (URL: https://www.dhsprogram.com/data/available-datasets.cfm) following their data obtaining procedure. However, no formal ethical clearance was obtained because the study involved secondary analysis of publically available data.

## Results

A total of 3071 children aged 0–59 months were included in the study. Stunting (44.4%) was the most common nutritional abnormality observed in this study followed by underweight (29.4%) and wasting (10.7%) (Table 2). Background characteristics of studied children are presented in Table 3. Briefly, mean age of the children was 2.1 years (SD 1.4) of which majority were males (50.7%), lived in rural areas (56.7%) and had mothers with no formal education (52.3%). Only 20.4% of the children had mothers with secondary education and 79.1% of the interviewed mothers were not working. About 20.6% of the children were from poorest households, whereas, 19.9% of the children were from richest households. According to the maternal characteristics, 41.7% of children had mothers who were married before 18 years of age, 62.3% had mothers who were consanguineously married and 32.9% had mothers who had given birth to more than 5 children. About 22.3% of the children had mothers who did not visit any antenatal clinic during pregnancy, 28.0% had mothers who did not have access to any source of information and 21.9% of the children had diarrhea in last two weeks before the survey was conducted.

**Table 3:** Socio-demographic characteristics of Pakistani children in the age range of < 5 years (*n* = 3071)

Variables	Total		Stunted	Underweight	Wasted
Maternal factors	n	%	(%)	(%)	(%)
Residence					
Urban	1330	43.3	39.9	21.4	9.8
Rural	1741	56.7	48.8	30.5	10.9
Mother’s educational level					
No education	1606	52.3	55.0	34.0	12.3
Primary	497	16.2	45.7	26.8	8.9
Secondary	626	20.4	29.1	15.7	8.3
Higher	342	11.1	25.7	11.4	7.6
Wealth index					
Poorest	634	20.6	62.3	37.9	12.8
Poorer	613	20.0	54.8	32.8	11.1
Middle	554	18.0	44.0	25.8	10.6
Richer	658	21.4	38.8	20.7	8.8
Richest	612	19.9	24.5	15.7	8.7
Mother’s employment status (*n* = 3064)					
Non-Working	2423	79.1	43.0	24.8	10.4
Working	641	20.9	52.7	33.4	10.3
Mother’s age at marriage (years)					
< 18	1281	41.7	52.1	31.9	11.0
≥ 18	1790	58.3	39.8	22.7	9.9
Parity					
1–4	2060	67.1	40.7	26.4	10.1
5+	1011	32.9	53.5	26.9	10.9
Mother’s access to information					
No	859	28.0	54.1	32.5	12.5
Yes	2212	72.0	41.1	24.3	9.6
Consanguineous marriage					
No	1159	37.7	38.4	21.0	9.8
Yes	1912	62.3	48.9	30.0	10.7
Mother’s height (cm) (*n* = 3064)					
Short Stature (< 145 cm)	132	4.3	70.5	50.0	11.4
Normal (≥ 145 cm)	2924	95.7	43.9	25.6	10.4
Mother’s BMI (kg/m^2^) (*n* = 3061)					
Underweight (<18.5 kg/m^2^)	364	11.9	58.5	45.3	15.7
Normal (18.5–24.9 kg/m^2^)	1657	54.3	45.6	28.2	11.1
Overweight (25.0–29.9 kg/m^2^)	696	22.8	43.1	18.7	8.5
Obese (≥ 30 kg/m^2^)	336	11.0	32.1	16.1	5.1
Child Factors					
Sex of child					
Male	1558	50.7	46.8	28.2	11.6
Female	1513	49.3	43.0	24.9	9.2
Child size at birth (*n* = 3066)					
Smaller than average	542	17.7	51.8	34.7	12.7
Average	2280	74.4	44.7	25.4	10.0
Larger than average	244	8.0	32.0	18.4	8.6
Antenatal clinic visits (*n* = 1967)					
None	439	22.3	57.4	33.0	13.9
1–3	716	36.4	44.4	30.0	12.7
3+	812	41.3	33.1	20.0	10.8
Recent diarrheal incidence (last two weeks) (*n* = 3076)					
No	2395	78.1	44.8	25.4	9.6
Yes	672	21.9	45.7	31.0	13.4
Breastfeeding status (n = 3066)					
Ever breastfeed	2016	65.8	48.5	25.3	8.0
Never breastfed	84	2.7	33.3	19.0	8.3
Still breastfeeding	966	31.5	38.6	29.8	15.4
Age of Child (months)					
0 (< 12 months)	580	18.9	25.0	24.0	15.2
1 (12-23 months)	551	17.9	45.2	28.7	15.6
2 (24-35 months)	652	21.2	54.3	28.5	8.1
3 (36-47 months)	633	20.6	50.1	25.6	7.7
4 (48–59 months)	655	21.3	48.1	26.1	6.6
Region					
Punjab	1018	33.1	37.7	23.8	8.3
Sindh	704	22.9	55.1	40.1	12.9
Khyber Pakhtunkhwa	541	17.6	37.9	24.0	11.5
Balochistan	292	9.5	78.8	33.6	12.7
Gilgit Baltistan	301	9.8	41.5	12.0	7.0
Islamabad capital territory (ICT)	215	7.0	22.3	13.0	11.2

### Determinants of stunting

Univariate analysis (Model 0) indicated that children born to mothers that lived in rural areas (cOR = 1.57, 95%CI 1.25–1.96), had no education (cOR = 4.66, 95%CI 3.10–7.00), had married consanguineously (cOR = 1.64, 95%CI 1.31–2.05) and had the poorest wealth index (cOR = 5.41, 95%CI 3.91–7.48) were more likely to be stunted. Mother’s height was significantly associated with child stunting as children whose mothers had short stature (< 145 cm) were more likely to be stunted (cOR = 3.05, 95%CI 1.68–5.55). This association remained the same after the adjustment of all other factors in Model 1 and 2. However, children whose mothers were aged ≥18 years at marriage (aOR = 0.76, 95%CI 0.59–0.99) and children whose mothers had visited antenatal clinics more than 3 times during pregnancy (aOR = 0.61, 95%CI 0.38–0.98) were less likely to be stunted. Children within the age group of 24–35 months (aOR = 3.65, 95%CI 2.23–5.95) and children that were of smaller than average size at the time of birth (aOR = 1.48, 95%CI 1.02–2.16) were more likely to be stunted (Table 4).

**Table 4:** Univariate and multivariate analysis of Stunting (Height-for-age index)

Variables	Model 0 (n = 3071)		Model 1 (*n* = 3046)		Model 2 (*n* = 1950)	
	cOR (95% CI)	*p*-value	aOR (95% CI)	*p*-value	aOR (95% CI)	*p*-value
Maternal factors						
Residence						
Urban	1		1		1	
Rural	1.57 (1.25–1.96)	< 0.01	0.70 (0.53–0.92)	0.01	0.67 (0.48–0.92)	0.01
Mother’s educational level						
Higher	1		1		1	
Secondary	1.28 (0.80–2.00)	0.28	0.98 (0.62–1.55)	0.94	0.93 (0.56–1.56)	0.80
Primary	3.21 (1.96–5.24)	< 0.01	1.95 (1.15–3.28)	0.01	1.40 (0.79–2.50)	0.24
No education	4.66 (3.10–7.00)	< 0.01	2.11 (1.29–3.43)	< 0.01	1.59 (0.88–2.88)	0.12
Wealth index						
Richest	1		1		1	
Richer	2.01 (1.39–2.91)	< 0.01	1.70 (1.14–2.52)	< 0.01	1.23 (0.76–2.00)	0.41
Middle	2.22 (1.55–3.17)	< 0.01	1.60 (1.02–2.53)	0.04	1.20 (0.66–2.17)	0.53
Poorer	4.16 (2.93–5.88)	< 0.01	2.64 (1.59–4.39)	< 0.01	1.72 (0.93–3.18)	0.08
Poorest	5.41 (3.91–7.48)	< 0.01	3.22 (1.86–5.59)	< 0.01	2.09 (1.05–4.14)	0.03
Mother’s employment status						
Non-working	1		1		1	
Working	1.45 (1.18–1.77)	< 0.01	0.94 (0.77–1.16)	0.61	0.92 (0.73–1.16)	0.49
Mother’s age at marriage (years)						
< 18	1		1		1	
>18	0.52 (0.42–0.64)	< 0.01	0.79 (0.63–0.99)	0.04	0.76 (0.59–0.99)	0.04
Parity						
1–4	1		1		1	
5+	1.40 (1.10–1.78)	< 0.01	0.99 (0.76–1.29)	0.97	1.06 (0.78–1.45)	0.67
Mother’s access to information						
No	1		1		1	
Yes	0.53 (0.39–0.71)	< 0.01	0.98 (0.66–1.45)	0.94	1.02 (0.68–1.53)	0.91
Consanguineous marriage						
No	1		1		1	
Yes	1.64 (1.31–2.05)	< 0.01	1.22 (0.96–1.54)	0.09	1.19 (0.91–1.55)	0.20
Mother’s height (cm)						
Normal (≥145 cm)	1		1		1	
Short (< 145 cm)	3.05 (1.68–5.55)	< 0.01	2.37 (1.34–4.23)	< 0.01	1.91 (1.04–3.51)	0.03
Mother’s BMI (kg/m^2^)						
Obese (≥30)	1		1		1	
Overweight (25.0–29.9)	1.17(0.80–1.70)	0.41	1.11 (0.77–1.60)	0.56	1.36 (0.87–2.12)	0.17
Normal (18.5–24.9)	1.67 (1.13–2.47)	< 0.01	1.20 (0.81–1.77)	0.35	1.34 (0.87–2.06)	0.17
Underweight (< 18.5)	2.39(1.47–3.88)	< 0.01	1.42 (0.79–2.52)	0.23	1.72 (0.96–3.03)	0.06
Child Factors						
Sex of child						
Female	1				1	
Male	1.28 (1.05–1.56)	0.01			1.47 (1.14-1.89)	< 0.01
Age of Child (months)						
0 (< 12)	1				1	
1 (12–23)	2.33 (1.62–3.37)	< 0.01			2.56 (1.67–3.91)	< 0.01
2 (24–35)	2.92 (2.17–3.93)	< 0.01			3.65 (2.23–5.95)	< 0.01
3 (36–47)	2.42 (1.76–3.32)	< 0.01			3.61 (2.05–6.25)	< 0.01
4 (48–59)	2.13 (1.54–2.95)	< 0.01			2.28 (1.16–4.46)	0.01
Child size at birth						
Average	1				1	
Smaller than average	1.56 (1.21–2.01)	< 0.01			1.48 (1.02–2.16)	0.03
Larger than average	0.54 (0.34–0.86)	0.01			0.55 (0.33–092)	0.02
Antenatal clinic visits						
None	1				1	
1–3	0.57 (0.39–0.84)	< 0.01			0.68 (0.45–1.00)	0.05
3+	0.35 (0.24–0.51)	< 0.01			0.61 (0.38–0.98)	0.04
Recent diarrheal incidence						
No	1				1	
Yes	1.14 (0.90–1.45)	0.26			1.06 (0.79–1.40)	0.67
Breastfeeding status						
Never breastfed	1				1	
Ever breastfed	1.49 (0.72–3.05)	0.27			0.94 (0.37–2.43)	0.91
Still breastfeeding	1.19 (0.58–2.45)	0.62			1.34 (0.50–3.55)	0.55

Model 0 = Univariate Analysis (cOR: Crude Odds Ratio)

Model 1 = Adjusted with Residence, mother’s education level, wealth index, mother’s employment status, mother’s age at marriage, parity, mother’s access to information, consanguineous marriage, mother’s height and mother’s BMI (aOR: Adjusted Odds Ratio)

Model 2 = Model 1 + Sex of child, age of child, child’s size at birth, antenatal clinic visits, recent diarrheal incidence, and breastfeeding status (aOR: Adjusted Odds Ratio)

### Determinants of underweight

Univariate analysis (Model 0) indicated that rural residence (cOR = 1.52, 95%CI 1.17–1.97), poorest wealth index (cOR = 5.01, 95%CI 3.47–7.22), mother’s employment status (cOR = 1.38, 95%CI 1.04–1.81), consanguineous marriage (cOR = 1.74, 95%CI 1.36–2.23) and male sex of the child (cOR = 1.30, 95%CI 1.03–1.63) were risk factors for child’s low weight-for-age status. Multivariate analysis with the addition of maternal and child related factors (Model 1 and Model 2) indicated that mother’s educational level (aOR = 2.55, 95%CI 1.26–5.17), child’s small size at birth (aOR = 1.67, 95%CI 1.14–2.45) and mother’s nutritional status were significantly associated with child’s low weight-for-age status. Children whose mothers were short in height (aOR = 2.31, 95%CI 1.34–3.98) and had a BMI < 18.5 (aOR = 1.78, 95%CI 1.00–3.17) were more likely be underweight (Table 5).

**Table 5:** Univariate and multivariate analysis of Underweight (Weight-for-age index)

Variables	Model 0 (*n* = 3071)		Model 1 (*n* = 3046)		Model 2 (*n* = 1950)	
	cOR (95% CI)	*p*-value	aOR (95% CI)	*p*-value	aOR (95% CI)	*p*-value
Maternal factors						
Residence						
Urban	1		1		1	
Rural	1.52 (1.17–1.97)	< 0.01	0.69 (0.49–0.99)	0.04	0.80 (0.56–1.16)	0.25
Mother’s educational level						
Higher	1		1		1	
Secondary	1.64 (0.96–2.80)	0.06	1.43 (0.81–2.50)	0.21	1.24 (0.66–2.34)	0.51
Primary	3.42 (1.82–6.40)	< 0.01	2.59 (1.26–5.31)	< 0.01	1.75 (0.84–3.72)	0.14
No education	5.55 (3.51–8.77)	< 0.01	3.18 (1.76–5.74)	< 0.01	2.55 (1.26–5.17)	< 0.01
Wealth index						
Richest	1		1		1	
Richer	1.57 (0.99–2.49)	0.05	1.18 (0.74–1.87)	0.49	0.86 (0.52–1.43)	0.57
Middle	1.90 (1.26–2.87)	< 0.01	1.14 (0.68–1.90)	0.61	0.97 (0.56–1.71)	0.93
Poorer	2.80 (1.95–4.03)	< 0.01	1.45 (0.86–2.51)	0.17	1.14 (0.63–2.05)	0.65
Poorest	5.01 (3.47–7.22)	< 0.01	2.41 (1.32–4.39)	< 0.01	1.78 (0.95–3.34)	0.06
Mother’s employment status						
Non-working	1		1		1	
Working	1.38 (1.04–1.81)	0.02	0.87 (0.66–1.15)	0.33	0.96 (0.67–1.38)	0.85
Mother’s age at marriage (years)						
<18	1		1		1	
>18	0.52 (0.40–0.66)	< 0.01	0.76 (0.61–0.96)	0.02	0.78 (0.57–1.04)	0.09
Parity						
1–4	1		1		1	
5+	0.96 (0.76–1.20)	0.73	0.68 (0.51–0.88)	< 0.01	0.73 (0.50–1.05)	0.09
Mother’s access to information						
No	1		1		1	
Yes	0.55 (0.41–0.71)	< 0.01	0.94 (0.69–1.29)	0.73	0.94 (0.65–1.36)	0.75
Consanguineous marriage						
No	1		1		1	
Yes	1.74 (1.36–2.23)	< 0.01	1.27 (0.99–1.63)	0.05	1.14 (0.86–1.50)	0.34
Mother’s height (cm)						
Normal (≥145 cm)	1		1		1	
Short (< 145 cm)	2.87 (1.69–4.89)	< 0.01	2.42 (1.48–3.92)	< 0.01	2.31 (1.34–3.98)	< 0.01
Mother’s BMI (kg/m^2^)						
Obese (≥30)	1		1		1	
Overweight (25.0–29.9)	0.87 (0.57–1.33)	0.53	0.82 (0.51–1.31)	0.41	0.79 (0.45–1.36)	0.39
Normal (18.5–24.9)	1.94 (1.25–3.01)	< 0.01	1.44 (0.89–2.33)	0.13	1.30 (0.76–2.22)	0.33
Underweight (<18.5)	3.40 (2.19–5.26)	< 0.01	2.04 (1.19–3.51)	< 0.01	1.78 (1.00–3.17)	0.04
Child Factors						
Sex of child						
Female	1				1	
Male	1.30 (1.03–1.63)	0.02			1.24 (0.94–1.63)	0.12
Age of Child (months)						
0 (< 12)	1				1	
1 (12–23)	1.12 (0.77–1.64)	0.54			1.31 (0.88–1.94)	0.17
2 (24–35)	0.98 (0.72–1.33)	0.91			1.18 (0.70–1.96)	0.52
3 (36–47)	0.96 (0.67–1.39)	0.86			1.69 (0.89–3.21)	0.10
4 (48–59)	0.89 (0.62–1.28)	0.54			1.65 (0.80–3.35)	0.17
Child size at birth						
Average	1				1	
Smaller than average	1.69 (1.28–2.21)	< 0.01			1.67 (1.14–2.45)	<0.01
Larger than average	0.66 (0.41–1.06)	0.09			0.97 (0.53–1.76)	0.94
Antenatal clinic visits						
None	1				1	
1–3	0.81 (0.56–1.17)	0.26			1.03 (0.70–1.51)	0.78
3+	0.46 (0.30–0.71)	< 0.01			0.91 (0.57–1.46)	0.72
Recent diarrheal incidence						
No	1				1	
Yes	1.27 (0.99–1.62)	0.05			1.15 (0.85–1.55)	0.29
Breastfeeding status						
Never breastfed	1				1	
Ever breastfed	1.51 (0.63–3.59)	0.35			1.03 (0.43–2.46)	0.94
Still breastfeeding	2.08 (0.88–4.88)	0.09			1.71 (0.72–4.05)	0.22

Model 0 = Univariate Analysis (cOR: Crude Odds Ratio)

Model 1 = Adjusted with Residence, mother’s education level, wealth index, mother’s employment status, mother’s age at marriage, parity, mother’s access to information, consanguineous marriage, mother’s height and mother’s BMI (aOR: Adjusted Odds Ratio)

Model 2 = Model 1 + Sex of child, Age of child, child’s size at birth, antenatal clinic visits, recent diarrheal incidence, and breastfeeding status (aOR: Adjusted Odds Ratio)

### Determinants of wasting

Multivariate analysis with the addition of maternal and child related factors (Model 1 and Model 2) indicated that children whose mothers had no education were more likely to be wasted (aOR = 3.61, 95%CI 1.33–9.82). Similarly, children whose mothers had a BMI < 18.5 were more likely to be wasted as compared to the children of obese mothers (aOR = 2.79, 95%CI 1.15–6.73) (Table 6).

**Table 6:** Univariate and multivariate analysis of Wasting (Weight-for-height index)

Variables	Model 0 (*n* = 2863)		Model 1 (*n* = 2840)		Model 2 (*n* = 1810)	
	cOR (95% CI)	*p*-value	aOR (95% CI)	*p*-value	aOR (95% CI)	*p*-value
Maternal factors						
Residence						
Urban	1		1		1	
Rural	1.13 (0.75–1.72)	0.54	0.70 (0.42–1.14)	0.15	0.85 (0.49–1.46)	0.55
Mother’s educational level						
Higher	1		1		1	
Secondary	1.31 (0.56–3.15)	0.54	1.25 (0.52–3.27)	0.62	2.14 (0.87–5.85)	0.09
Primary	1.55 (0.76–3.25)	0.23	1.55 (0.69–3.44)	0.28	2.41 (0.89–7.15)	0.07
No education	2.46 (1.15–5.28)	0.02	1.87 (0.82–4.21)	0.13	3.61 (1.33–9.82)	< 0.01
Wealth index						
Richest	1		1		1	
Richer	0.95 (0.60–1.56)	0.83	0.82 (0.45–1.49)	0.52	0.73 (0.34–1.58)	0.43
Middle	1.15 (0.70–1.98)	0.59	0.90 (0.44–1.84)	0.78	0.82 (0.35–1.93)	0.66
Poorer	1.21 (0.69–2.10)	0.49	0.87 (0.39–1.92)	0.73	0.72 (0.27–1.88)	0.50
Poorest	2.36 (1.40–3.98)	< 0.01	1.69 (0.78–3.66)	0.18	1.65 (0.62–4.16)	0.32
Mother’s employment status (n = 3064)						
Non-working	1		1		1	
Working	0.94 (0.61–1.50)	0.81	0.66 (0.49–0.99)	0.04	0.72 (0.43–1.23)	0.24
Mother’s age at marriage (years)						
<18	1		1		1	
>18	0.77 (0.52–1.14)	0.19	1.03 (0.74–1.45)	0.85	0.93 (0.58–1.42)	0.69
Parity						
1–4	1		1		1	
5+	1.07 (0.78–1.45)	0.68	0.96 (0.66–1.41)	0.85	1.10 (0.66–1.83)	0.67
Mother’s access to information						
No	1		1		1	
Yes	0.58 (0.40–0.83)	< 0.01	0.78 (0.55–1.09)	0.14	0.94 (0.67–1.34)	0.76
Consanguineous marriage						
No	1		1		1	
Yes	1.20 (0.84–1.71)	0.31	1.01 (0.71–1.41)	0.95	0.96 (0.65–1.41)	0.83
Mother’s height (cm)						
Normal (≥ 145 cm)	1		1		1	
Short (< 145 cm)	1.46 (0.74–2.76)	0.24	1.30 (0.68–2.51)	0.42	1.29 (0.54–3.04)	0.55
Mother’s BMI (kg/m^2^)						
Obese (≥30)	1		1		1	
Overweight (25.0–29.9)	1.08 (0.50–2.25)	0.82	1.05 (0.48–2.28)	0.89	1.27 (0.51–3.15)	0.60
Normal (18.5–24.9)	1.85 (0.94–3.64)	0.07	1.61 (0.83–3.11)	0.15	1.77 (0.74–4.06)	0.19
Underweight (<18.5)	2.85 (1.43–5.68)	< 0.01	2.39 (1.17–4.87)	0.01	2.79 (1.15–6.73)	0.02
Child Factors						
Sex of child						
Female	1				1	
Male	1.15 (0.84–1.56)	0.38			1.25 (0.87–1.79)	0.21
Age of Child (months)						
0 (< 12)	1				1	
1 (12–23)	0.85 (0.57–1.25)	0.41			0.82 (0.52–1.30)	0.41
2 (24–35)	0.35 (0.23–0.54)	< 0.01			0.26 (0.12–0.59)	< 0.01
3 (36–47)	0.46 (0.23–0.98)	0.04			0.50 (0.18–1.41)	0.19
4 (48–59)	0.23 (0.13–0.41)	< 0.01			0.25 (0.09–0.74)	0.01
Child size at birth						
Average	1				1	
Small	1.35 (0.89–2.03)	0.14			1.24 (0.78–1.96)	0.36
Large	0.66 (0.34–1.32)	0.25			0.78 (0.32–1.89)	0.59
Antenatal clinic visits						
None	1				1	
1–3	0.67 (0.39–1.14)	0.14			0.74 (0.46–1.16)	0.19
3+	0.60 (0.29–1.17)	0.13			0.92 (0.51–1.67)	0.80
Recent diarrheal incidence						
No	1				1	
Yes	1.43 (1.02–1.98)	0.03			1.11 (0.74–1.64)	0.61
Breastfeeding status						
Never breastfed	1				1	
Ever breastfed	1.14 (0.36–3.78)	0.82			1.16 (0.32–3.87)	0.85
Still breastfeeding	2.42 (0.77–7.56)	0.12			0.82 (0.24–2.76)	0.74

Model 0 = Univariate Analysis (cOR: Crude Odds Ratio)

Model 1 = Adjusted with Residence, mother’s education level, wealth index, mother’s employment status, mother’s age at marriage, parity, mother’s access to information, consanguineous marriage, mother’s height and mother’s BMI (aOR: Adjusted Odds Ratio)

Model 2 = Model 1 + Sex of child, age of child, child’s size at birth, antenatal clinic visits, recent diarrheal incidence, and breastfeeding status (aOR: Adjusted Odds Ratio)

## Discussion

This study presents the risk factors associated with child malnutrition in terms of stunting, underweight and wasting among under-five Pakistani children using the 2012–2013 PDHS data. Our study showed that place of residence, wealth index, BMI of mother, mothers’ age at marriage, child size at birth and antenatal clinic visits have significant independent association with child nutritional status. The magnitude of malnutrition observed in this study reinforced the need to take actions to improve the nutritional status of children in Pakistan. The most common form of malnutrition among the studied population (*n* = 3071) was stunting (44.4%) followed by underweight (29.4%) and wasting (10.7%).

In Pakistan, the prevalence of stunting in children < 5 years is very high (44%) as compared to other neighboring regional countries like Bangladesh (36%) and Nepal (35.8%) [15, 29]. The magnitude of stunting was only reduced by 5% when compared with the country’s previous demographic health survey conducted in 1990–1991 [30]. This showed that the prevalence of stunting in Pakistan remains consistently high over past 20 years. This study highlights both maternal and child factors, associated with stunting, that should be thoroughly investigated for implementing appropriate interventions to reduce the burden of stunting in Pakistan.

Our study showed that among the various factors related to stunting, household wealth index was most significantly associated. The odds of being stunted were substantially higher among children with lowest socioeconomic background. This finding was in line with previous cross-sectional studies carried out in countries such as Bangladesh, Nepal and Peru [15, 16, 31]. Socio-economic status has an impact on household food security and subsequently the growth of children [19]. Children from poor households have limited access to food and health services, which makes them more susceptible to growth failures [19]. Therefore, policies which are focused on poverty alleviation and improving the nutritional status of poorer children (either through cash or food support program [32, 33]) are needed to address the under-five malnutrition issue in Pakistan.

Mother’s nutritional status is associated with child’s nutritional state [17]. The risk of wasting and underweight was higher in those children whose mothers’ BMI were below normal (<18.5 kg/m^2^). Maternal height has been used as a marker to assess the intergenerational health linkages between a mother and her offspring. Previous evidence had shown several adverse health related consequences, including the nutritional outcomes, in children of mothers of low height (or short stature) [34, 35]. Thus, mother’s height can be used as predictor of child’s nutritional status [36]. In our study, children of short stature mothers (height = < 145 cm) were found more likely to be stunted and underweight. These findings of the effect of mothers’ anthropometry (BMI and low height) on children were in accordance with previous studies [17, 34, 37, 38]. Since mother’s nutritional and health status has critical importance in early child growth and development [39], mother’s nutritional status should be considered when making policies for reducing the child malnutrition. Emphasis on reproductive health of adolescent females is now being considered very important in developing countries, particularly for the outcome of improved nutrition of children < 5 years of age [39, 40]. There have been efforts in the past to address nutritional issues among young girls, pregnant and lactating women in country with different nutrition programs such as “Tawana Pakistan Project” [40]. However, the female adolescent nutrition in Pakistan is very recent and needs a greater focus.

Our analysis showed that children of mothers with no formal education were more prone to be acutely malnourished (wasted and underweight) as compared to children of educated mothers. The association found in this study between maternal education, wasting and underweight in children is consistent with several previous studies [19, 41, 42]. Educated mothers are well informed about the nutritional and health needs of their children and hence prefer to use better hygiene and sanitation facilities. Moreover, they make comparative choices of available health services over traditional practices for improved healthcare of their children [24].

Early marriage (before 18 years) increased the risk of child stunting and underweight. Young women are at an age when they still need to provide for their own growth and developmental needs. Pregnancy in such women increases the drain of their already low reserve of nutrients and thus increases the probability of delivering low birth weight infants [43]. These mothers are also at risk to inadequately breastfeed their infants due to low milk supply which results in undernourished children [43, 44]. However, future studies and large scale national surveys may be needed to record the age of the first pregnancy, a more important factor than age of marriage, to investigate relationships between mother’s age and child nutritional status.

In our study several maternal factors are found to be associated with child malnutrition (such as mother’s age at marriage, education and nutritional status). These findings indicate that nutrition interventional programs should, therefore, encompass maternal socio-demographic factors for the betterment of nutritional status of children, below the age of five years, in Pakistan. Additionally, there is a need for national nutritional policy, focused on improving both the maternal nutritional status (through adequate food and micronutrients supplementation) and the better care for infants and young children [45].

Our study found that children who lived in urban areas of Pakistan were more vulnerable to become stunted. These results are consistent with the previous regional studies carried out in Bangladesh and Iran [15, 46]. On the contrary, several studies conducted in developing countries (such as Nepal, Bangladesh, Malawi and Nigeria etc.) have identified that children settled in rural areas, are at higher risk to be malnourished [47, 48]. In Pakistan, the high trend of stunting in urban areas may be due to the rapid urbanization of people migrating from rural areas for work and better living conditions. The increased incidence of stunting in urban areas could also be reflective of the dietary choices and life style of urban population [49].

Our results showed that male children were more likely to be stunted as compared to female children. The finding was consistent with the previous research reported that male children are more vulnerable to develop malnutrition because they require comparatively more calories for growth and development [46, 50]. One of the reasons for low caloric intake in children is their low socioeconomic status as observed in our study. This may lead to increased susceptibility to stunting among male children.

Our study also found that the odds of stunting significantly increased with child’s age. Children within the age group of 12–59 months were more likely to be stunted compared to younger children (less than 12 months). Similar findings were also reported by other studies conducted in different parts of the world [16, 51]. The increase in child stunting with age, stresses the need of proper and timely initiation of supplementary feeding to meet the growing nutritional requirements of the children. Additionally, the risk of wasting was significantly lower in children > 23 months as compared to children < 12 months. This data is consistent with previous findings that showed a decrease in wasting with child’s age and thus, may be associated with the inclusion of other food items along with breast milk in children’s diet after 6 months of age (15,17).

Among the study population, children whose mother visited antenatal clinics more frequently were less likely to have chronic malnutrition (stunting). Antenatal visits are considered to be an indicator of access to health care services and maternal health seeking behavior. This may have indirect influence on child’s health both in the short and long term [52]. Previous studies have also showed that access to health services are positively correlated with child’s nutritional status [46, 53]. This finding has important policy implications associated with access to either free or at least affordable health services to help in reducing the burden of childhood malnutrition. Additionally, in Pakistan, there is a need to increase the availability of quality primary health care facilities in order to improve the child health status [54].

Perceived child size at birth significantly determined the nutritional status of the child [55], as low birth weight is considered to be an indicator of restricted intrauterine growth [56]. Our study found that children born with smaller than the average birth weight were more likely to be underweight, whereas those born with larger than average weight were less likely to be stunted. These findings were consistent with previous studies which showed that low birth weight infants have significantly higher odds of being stunted and underweight later in life due to inadequate fetal nutrition [57–59].

The strengths of our study includes the use of nutritional data from the most recent population based representative survey (PDHS-2012-13) to assess the malnutrition in Pakistani children aged 0–59 months. Furthermore, the survey had a large sample size and high response rate (93%).The study also had certain limitations including the study design which was cross-sectional and hence, difficult to establish causal relationships between different variables. Moreover, micronutrient consumption and other dietary factors directly related to the nutritional status of children were not available.

## Conclusion

In conclusion, our study found that both maternal and child related factors are associated with malnutrition in Pakistani children and most of them are preventable. In order to reduce the burden of early malnutrition in the country, strategies which are focused on poverty eradication, improvement of both mother’s educational and nutritional level and accessibility to basic health care facilities are needed. Furthermore, interventions that can address these factors are required such as community based education and targeted nutritional interventions.

## Abbreviations

AJK: Azad Jammu and Kashmir
aOR: Adjusted odds ratio
BMI: Body mass index
CI: Confidence intervals
cOR: Crude odds ratio
DHS: Demographic and Health Surveys
FATA: Federally Administrated Tribal Areas
ICT: Islamabad Capital Territory
NIPS: National Institute of Population Studies
PDHS: Pakistan Demographic and Health Survey
SD: Standard deviation
USAID: United States Agency for International Development (USAID)
WHO: World Health Organization

## References

[1] WHO (2013). Guideline: Updates on the management of severe acute malnutrition in infants and children.

[2] Bomela NJ (2009). Social, economic, health and environmental determinants of child nutritional status in three central Asian republics. Public Health Nutr.

[3] Pelletier DL, Frongillo EA, Schroeder DG, Habichit JP (1995). The effects of malnutrition on child mortality in developing countries. Bull World Health Organ.

[4] Pelletier DL, Frongillo EA (2003). Changes in child survival are strongly associated with changes in malnutrition in developing countries. J Nutr.

[5] De Onis M, Blössner M (2003). The World Health Organization global database on child growth and malnutrition: methodology and applications. Int J Epidemiol.

[6] Khara T, Mwangome M, Ngari M, Dolan C (2018). Children concurrently wasted and stunted: a meta-analysis of prevalence data of children 6–59 months from 84 countries. Matern Child Nutr.

[7] Liu L, Oza S, Hogan D, Perin J, Rudan I, Lawn JE (2015). Global, regional, and national causes of child mortality in 2000–13, with projections to inform post–2015 priorities: an updated systematic analysis. Lancet.

[8] World Health Organization (2016). The double burden of malnutrition: policy brief.

[9] Black RE, Victora CG, Walker SP (2013). Maternal and child undernutrition and overweight in low-income and middle-income countries. Lancet.

[10] The United Nations Children’s Fund. World Health Organization. World Bank. UNICEF (2015). WHO—The World Bank Child Malnutrition Database: Estimates for 2015 and Launch of Interactive Data Dashboards.

[11] Headey D, Hoddinott J, Park S (2016). Drivers of nutritional change in four south Asian countries: a dynamic observational analysis. Matern Child Nutr.

[12] Bhutta ZA, Hafeez A, Rizvi A, Ali N, Khan A, Ahmad F (2013). Reproductive, maternal, newborn, and child health in Pakistan: challenges and opportunities. Lancet..

[13] UNICEF (2016). The state of the World’s children 2016: a fair chance for every child, United Nations, New York.

[14] Bhutta ZA, Soofi SB, Zaidi SSH, Habib A (2011). Pakistan National Nutrition Survey, 2011.

[15] Das S, Gulshan J (2017). Different forms of malnutrition among under five children in Bangladesh: a cross sectional study on prevalence and determinants. BMC Nutr.

[16] Tiwari R, Ausman LM, Agho KE (2014). Determinants of stunting and severe stunting among under-fives: evidence from the 2011 Nepal demographic and health survey. BMC Pediatr.

[17] Akombi BJ, Agho KE, Merom D, Hall JJ, Renzaho AM (2017). Multilevel analysis of factors associated with wasting and underweight among children under-five years in Nigeria. Nutrients..

[18] Ersino G, Henry CJ, Zello GA (2016). Suboptimal feeding practices and high levels of undernutrition among infants and young children in the rural communities of Halaba and Zeway, Ethiopia. Food Nutr Bull.

[19] Chowdhury MR, Rahman MS, Khan MM, Mondal MN, Rahman MM, Billah B (2016). Risk factors for child malnutrition in Bangladesh: a multilevel analysis of a nationwide population-based survey. J Pediatr.

[20] Sand A, Kumar R, Shaikh BT, Somrongthong R, Hafeez A, Rai D (2018). Determinants of severe acute malnutrition among children under five years in a rural remote setting: a hospital based study from district Tharparkar-Sindh, Pakistan. Pak J Med Sci.

[21] Mustufa MA, Jamali AK, Sameen I, Burfat FM, Baloch MY, Baloch AH, Baloch GR, Lashari SK, Ayaz SM, Baloch MY (2017). Malnutrition and poor oral health status are major risks among primary school children at Lasbela, Balochistan, Pakistan. J Health Popul Nutr.

[22] Laghari ZA, Soomro AM, Tunio SA, Lashari K, Baloach FG, Baig NM, Bano S (2015). Malnutrition among children under five years in district Sanghar, Sindh, Pakistan. Gomal J Med Sci.

[23] Asim M, Nawaz Y (2018). Child malnutrition in Pakistan: evidence from literature. Children..

[24] Tariq J, Sajjad A, Zakar R, Zakar MZ, Fischer F (2018). Factors associated with undernutrition in children under the age of two years: secondary data analysis based on the Pakistan demographic and health survey 2012–2013. Nutrients..

[25] Di Cesare M, Bhatti Z, Soofi SB, Fortunato L, Ezzati M, Bhutta ZA (2015). Geographical and socioeconomic inequalities in women and children's nutritional status in Pakistan in 2011: an analysis of data from a nationally representative survey. Lancet Glob Health.

[26] National Institute of Population Studies (NIPS) [Pakistan] and ICF International (2013). Pakistan demographic and health survey 2012-13.

[27] World Health Organization (WHO), Multicenter Growth Reference Study Group (2006). WHO child growth standards: length/height-for-age, weight-for-length, weight-for-height and body mass index-forage: methods and development.

[28] Talukder A (2017). Factors associated with malnutrition among under-five children: illustration using Bangladesh demographic and health survey, 2014 data. Children..

[29] STATcompiler. The DHS Program STATcompiler. http://www.statcompiler.com. Accessed 25 Apr 2018.

[30] Arif GM, Nazir S, Satti MN, Farooq S. Child malnutrition in Pakistan: trends and determinants. Pak Inst Dev Econ. 2012. p. 1–8.

[31] Urke HB, Bull T, Mittelmark MB (2011). Socioeconomic status and chronic child malnutrition: wealth and maternal education matter more in the Peruvian Andes than nationally. Nutr Res.

[32] Fenn B, Sangrasi GM, Puett C, Trenouth L, Pietzsch S (2015). The REFANI Pakistan study—a cluster randomised controlled trial of the effectiveness and cost-effectiveness of cash-based transfer programmes on child nutrition status: study protocol. BMC Public Health.

[33] Kureishy S, Khan GN, Arrif S, Ashraf K, Cespedes A, Habib MA, Hussain I, Ullah A, Turab A, Ahmed I, Zaidi S (2017). A mixed methods study to assess the effectiveness of food-based interventions to prevent stunting among children under-five years in districts Thatta and Sujawal, Sindh Province, Pakistan: study protocol. BMC Public Health.

[34] Subramanian SV, Ackerson LK, Smith GD, John NA (2009). Association of maternal height with child mortality, anthropometric failure, and anemia in India. Jama..

[35] Addo OY, Stein AD, Fall CH, Gigante DP, Guntupalli AM, Horta BL, Kuzawa CW, Lee N, Norris SA, Prabhakaran P, Richter LM (2013). Maternal height and child growth patterns. J Pediatr.

[36] Felisbino-Mendes MS, Villamor E, Velasquez-Melendez G (2014). Association of maternal and child nutritional status in Brazil: a population based cross-sectional study. PLoS One.

[37] Hasan MT, Soares Magalhães RJ, Williams GM, Mamun AA (2016). Long-term changes in childhood malnutrition are associated with long-term changes in maternal BMI: evidence from Bangladesh, 1996–2011, 2. Am J Clin Nutr.

[38] Aguayo VM, Nair R, Badgaiyan N, Krishna V (2016). Determinants of stunting and poor linear growth in children under 2 years of age in India: an in-depth analysis of Maharashtra's comprehensive nutrition survey. Matern Child Nutr.

[39] Negash C, Whiting SJ, Henry CJ, Belachew T, Hailemariam TG (2015). Association between maternal and child nutritional status in hula, rural southern Ethiopia: a cross sectional study. PLoS One.

[40] Badruddin SH, Agha A, Peermohamed H, Rafique G, Khan KS, Pappas G (2008). Tawana project-school nutrition program in Pakistan-its success, bottlenecks and lessons learned. Asia Pac J Clin Nutr.

[41] Mishra K, Kumar P, Basu S, Rai K, Aneja S (2014). Risk factors for severe acute malnutrition in children below 5 y of age in India: a case-control study. Indian J Pediatr.

[42] Chisti MJ, Hossain MI, Malek MA, Faruque AS, Ahmed T, Salam MA (2007). Characteristics of severely malnourished under-five children hospitalized with diarrhoea, and their policy implications. Acta Paediatr.

[43] Raj A, Saggurti N, Winter M, Labonte A, Decker MR, Balaiah D, Silverman JG (2010). The effect of maternal child marriage on morbidity and mortality of children under 5 in India: cross sectional study of a nationally representative sample. BMJ..

[44] King JC (2003). The risk of maternal nutritional depletion and poor outcomes increases in early or closely spaced pregnancies. J Nutr.

[45] Iqbal S, Zakar R, Zakar MZ, Fischer F (2017). Factors associated with infants’ and young children’s (6–23 months) dietary diversity in Pakistan: evidence from the demographic and health survey 2012–13. Nutr J.

[46] Kavosi E, Rostami ZH, Kavosi Z, Nasihatkon A, Moghadami M, Heidari M (2014). Prevalence and determinants of under-nutrition among children under six: a cross-sectional survey in Fars province, Iran. Int J health Policy Manag.

[47] Van de Poel E, O’Donnell O, Van Doorslaer E (2007). Are urban children really healthier? Evidence from 47 developing countries. Soc Sci Med.

[48] Srinivasan CS, Zanello G, Shankar B (2013). Rural-urban disparities in child nutrition in Bangladesh and Nepal. BMC Public Health.

[49] Arif GM, Hamid S (2009). Urbanization, city growth and quality of life in Pakistan. Eur J Soc Sci.

[50] Demissie S, Worku A (2013). Magnitude and factors associated with malnutrition in children 6-59 months of age in pastoral community of Dollo ado district, Somali region, Ethiopia. Sci J Public Health.

[51] Darteh EK, Acquah E, Kumi-Kyereme A (2014). Correlates of stunting among children in Ghana. BMC Public Health.

[52] Hamel C, Enne J, Omer K, Ayara N, Yarima Y, Cockcroft A, Andersson N (2015). Childhood malnutrition is associated with maternal care during pregnancy and childbirth: a cross-sectional study in Bauchi and Cross River states, Nigeria. J Public Health Res.

[53] Kabubo-Mariara J, Ndenge GK, Mwabu DK (2008). Determinants of children’s nutritional status in Kenya: evidence from demographic and health surveys. J Afr Econ.

[54] Majrooh MA, Hasnain S, Akram J, Siddiqui A, Memon ZA (2014). Coverage and quality of antenatal care provided at primary health care facilities in the ‘Punjab’province of ‘Pakistan’. PLoS One.

[55] Rahman MS, Howlader T, Masud MS, Rahman ML (2016). Association of low-birth weight with malnutrition in children under five years in Bangladesh: do mother’s education, socio-economic status, and birth interval matter?. PLoS One.

[56] Sharma D, Shastri S, Sharma P (2016). Intrauterine growth restriction: antenatal and postnatal aspects. Clin Med Insights Pediatr.

[57] Brhane G, Regassa N (2014). Nutritional status of children under five years of age in Shire Indaselassie, North Ethiopia: examining the prevalence and risk factors. Kontakt..

[58] Ramakrishnan U, Neufeld LM, Flores R, Rivera J, Martorell R (2009). Multiple micronutrient supplementation during early childhood increases child size at 2 y of age only among high compliers. Am J Clin Nutr.

[59] Adhikari D, Khatri RB, Paudel YR, Poudyal AK (2017). Factors associated with underweight among under-five children in eastern Nepal: community-based cross-sectional study. Front Public Health.

